# Investigating associations between maternal stress, smoking and adverse birth outcomes: evidence from the All Our Families cohort

**DOI:** 10.1186/s12884-023-06029-y

**Published:** 2023-10-04

**Authors:** Shelby S. Yamamoto, Shahirose S. Premji, Vineet Saini, Sheila W. McDonald, Gian S. Jhangri

**Affiliations:** 1 School of Public Health, University of Alberta, 11405 87th Ave, Edmonton, AB T6G 1C9 Canada; 2 School of Nursing, Queen’s University, 92 Barrie Street, Kingston, ON K7L 3N6 Canada; 3 Alberta Health Services, Department of Research and Innovation, Provincial Population and Public Health, 10030 – 107 Street NW, Edmonton, AB T5J 3E4 Canada; 4 Cumming School of Medicine, Department of Community Health Sciences, University of Calgary, 3280 Hospital Drive NW, Calgary, AB T2N 4Z6 Canada

**Keywords:** Perinatal mental stress, Anxiety, Tobacco smoke, Preterm birth, Low birth weight, Interaction

## Abstract

**Background:**

Independently, active maternal and environmental tobacco smoke exposure and maternal stress have been linked to an increased risk of preterm birth and low birth weight. An understudied relationship is the potential for interactive effects between these risk factors.

**Methods:**

Data was obtained from the All Our Families cohort, a study of 3,388 pregnant women < 25 weeks gestation recruited from those receiving prenatal care in Calgary, Canada between May 2008 and December 2010. We investigated the joint effects of active maternal smoking, total smoke exposure (active maternal smoking plus environmental tobacco smoke) and prenatal stress (Perceived Stress Scale, Spielberger State-Trait Anxiety Inventory), measured at two time points (< 25 weeks and 34–36 weeks gestation), on preterm birth and low birth weight.

**Results:**

A marginally significant association was observed with the interaction active maternal smoking and Spielberger State-Trait Anxiety Inventory scores in relation to low birth weight, after imputation (aOR = 1.02, 95%CI: 1.00-1.03, p = 0.06). No significant joint effects of maternal stress and either active maternal smoking or total smoke exposure with preterm birth were observed. Active maternal smoking, total smoke exposure, Perceived Stress Scores, and Spielberger State-Trait Anxiety Inventory scores were independently associated with preterm birth and/or low birth weight.

**Conclusions:**

Findings indicate the role of independent effects of smoking and stress in terms of preterm birth and low birthweight. However, the etiology of preterm birth and low birth weight is complex and multifactorial. Further investigations of potential interactive effects may be useful in helping to identify women experiencing vulnerability and inform the development of targeted interventions.

## Background

Preterm birth (< 37 weeks gestational age) and low birth weight (< 2500 g) are ongoing issues globally, with rates increasing in many countries, despite our advances in knowledge and interventions aimed at known risk factors [1, 2]. In Canada, Alberta’s provincial preterm birth (9.2% 2019/2020) [3] and low birth weight (7.5%, 2019/2020) [3] rates are among the highest in the country, though the reasons for this are not immediately clear. Both preterm birth and low birth weight have been associated with increased risk of cardiovascular disease and diabetes in adulthood [4, 5].

Preterm birth and low birth weight have multifactorial etiologies, likely affected by a combination of genetic, biological, behavioral, environmental, social, and other factors [6, 7]. Among these are exposure to prenatal stress and tobacco smoke. There is evidence to suggest a role of for maternal stress in terms of adverse birth outcomes [8–10]. For example, Shapiro et al. [11] observed mostly consistent associations between perceived stress, pregnancy-related anxiety, and increased the risk of preterm birth, a finding also echoed in other reviews [9, 10]. Similarly, the risks associated with tobacco smoke exposure (active maternal and environmental tobacco smoke) during pregnancy have been well documented [12–15]. Smoke exposure has been linked to both preterm birth and low birth weight [12, 16, 17].

There is evidence to indicate that these independent relationships may be modified by other factors [7, 18–22]. Specifically, stress and smoke exposure may interact through pathways such as decreased uterine-placental blood flow, vasoconstriction of placental vessels or changes in immunologic functioning [23–25] Active maternal smoking may also be related to maternal stress. Maternal psychological stress was observed to be higher among pregnant women who smoked in a prospective study in Brazil [20]. Smoking may be a coping mechanism for pregnant women experiencing prenatal anxiety [26, 27] Stress during pregnancy could also act as a smoking cessation barrier [28, 29]. Data from the United States showed that though up to 45% of women are prompted to quit smoking during pregnancy, about 10% continue to smoke [29]. However, to date, little has been explored regarding potential interactions between stress and smoke exposure and its impact in terms of preterm birth and low birth weight risk. Disparities exist in terms of the population distribution of adverse birth outcomes that might be more fully explained by joint effects [11, 30]. Stress may lead to an increase in maternal smoking. Alternatively, smoking may also lower women’s perceived stress levels. Clinically, findings related to interactive effects could be important for designing more effective interventions that include a combination of targeted approaches (e.g. smoking cessation plus stress-reduction). In this study, we aimed to investigate the joint effects of total smoke exposure (active maternal and environmental tobacco smoke) and maternal stress (perceived stress and anxiety) on the risk of preterm birth and low birth weight and potential relationships between active maternal smoking and maternal stress among pregnant women in the All Our Families cohort.

## Methods

### Study population

All our Families (AOF) is a prospective cohort study of pregnant women in Calgary, Canada set up to investigate determinants of maternal, infant and child wellbeing and health service utilization during and after pregnancy [31]. The study recruited 3,388 pregnant women < 25 weeks gestation undergoing prenatal care between May 2008 and December 2010 [31]. Women were asked to complete three mailed questionnaires - at recruitment (< 25 weeks gestation), 34–36 weeks gestation, and 4 months postpartum [31]. The study is also following up children at 1, 2, 3, 5, and 8 years postpartum [31]. Further detail about the recruitment and follow up is described elsewhere [31]. Data were collected on demographics, mental health, physical health, psychosocial factors, lifestyle, pregnancy history, health service utilization, parenting, pregnancy outcomes, and child development [31]. Ethics approval was obtained from Child Health Research Office and the Conjoint Health Research Ethics Board of the Faculties of Medicine, Nursing, and Kinesiology (University of Calgary) and the Affiliated Teaching Institutions [31].

This study examined two outcomes: preterm birth defined as < 37 weeks gestation and infant low birth weight defined as < 2,500 g among primiparous women. Exclusions included multiple births as this is an independent risk factor for poorer maternal mental health, preterm birth and low birth weight [32–34]. The primary exposures examined in this study included smoke exposure (active maternal, total smoke) and maternal stress. Active maternal smoking included those women who reported smoking during pregnancy (yes/no) in the survey. Total smoke exposure was defined as exposure to either active maternal or environmental (i.e., partner was reported as smoking in survey) tobacco smoke. Both active and environmental tobacco smoke exposure were assessed only at < 25 weeks gestation. Prenatal stress was measured at two time periods (< 25 weeks and 34–36 weeks gestation) using the Perceived Stress Scale (PSS) (possible range: 0 to 40) [35] and Spielberger State-Trait Anxiety Inventory (STAI) (possible range: 20 to 80) [36] tools. PSS and STAI scores were treated as continuous variables. Other potential confounders identified *a priori* from the literature and included in the models were: maternal age, derived from mother’s birthdate at the time the first maternal questionnaire was administered (< 25 weeks gestation); maternal highest level of education categorized into high school or less/some trade school, college, or university/graduated trade school, college, university/some graduate school, completed graduate school; household income grouped into <$40,000/$40,000 to <$60,000/$60,000 to <$80,000/$80,000 to <$100,000/≥$100,000; body mass index (BMI) categorized as underweight (< 18.5 kg/m^2^)/normal weight (18.5-<25.0 kg/m^2^)/overweight (25.0-<30.0 kg/m^2^)/obese (≥ 30.0 kg/m^2^); and parity (previous birth, no previous birth).

### Statistical analyses

A Generalized Estimating Equation logistic regression approach was used to account for the two stress measures assessed at < 25 weeks and 34–36 weeks gestation. To explore the hypotheses that maternal stress (PSS scores, STAI scores) may trigger maternal smoking behavior, we also conducted multiple linear regression. Adjusted models also included the potential confounders described above. Recognizing that prior preterm birth is an important predictor of subsequent adverse birth outcomes, [8] we conducted sensitivity analyses including only women who had previously given birth (multiparous) to assess the robustness of the results. Both prior history of preterm birth and prior history of low birth weight were also included in multiple imputation models. An *a priori* sample size of 3,241 was estimated for a multiplicative interaction, based on findings from a prior study examining cumulative psychosocial stress and late (34–36^6/7^ weeks) preterm birth (OR = 1.73, preterm birth rate = 7%, smoking during pregnancy = 15.2%, stress during pregnancy = 23.7%) using the same cohort data [37], the association of preterm birth with smoking during pregnancy (OR = 1.35) [38], and maternal smoking and serious psychological distress (OR = 2.37) [39].

We conducted full conditional specification multiple imputation, including auxiliary variables, for the outcomes preterm birth (10.8% missing), low birth weight (15.0% missing), total smoke exposure (11.7% missing), active total smoke exposure (13.1% missing), perceived stress scale scores for the first and second time point (2.1% and 14.1% missing, respectively), Spielberger State-Trait Anxiety Inventory scores for the first and second time point (4.4% and 8.3% missing, respectively), PSS-total smoke exposure interactions (15.5% missing), STAI-total smoke exposure interactions (15.2% missing) and parity (1.5% missing) [40]. Interactions were first calculated and then imputed to avoid biases [41]. We imputed 20 data sets. To assess potential departures from our missing at random assumption, we also performed sensitivity analyses. All analyses were performed using SAS 9.4.

## Results

### Descriptives

The All Our Families cohort included 3,387 women who completed at least one questionnaire [42]. The mean age of the women was 30.4 (95%CI: 30.3–30.6) years. The majority of women in the cohort reported their ethnicity as white/Caucasian (n = 2,408; 79%). The median gestational age was 39 weeks (IQR: 38–40) and the mean birthweight of infants was 3,366 g (95%CI: 3,346-3,386). There were 190 (6.9%) preterm births and 124 (4.7) low birth weight infants in the cohort. Slightly less than half (n = 1,431, 47.6%) of the infants were female.

Twenty-four percent of the women in the cohort were exposed to either active or environmental tobacco smoke during pregnancy (Table 1). Of these, 12% reported actively smoking during pregnancy and 39% reported smoking one or more cigarettes per day. Nearly 16% reported having a partner who smoked. Less than 1% reported smoking as permitted inside their homes. The median Perceived Stress Scale scores at < 25 (13, IQR: 9–18) and 34–36 (13, IQR: 8–17) weeks gestation were similar. Slightly higher Spielberger State Anxiety Inventory scores were recorded at 34–36 weeks gestation compared with < 25 weeks gestation.

**Table 1:** Maternal and infant characteristics of cohort participants

**Characteristics**		Preterm birth	Term birth	Low birth weight	Normal birth weight
		**n (%** ^**a**^ **)**	**n (%** ^**a**^ **)**	n (%^a^)	n (%^a^)
*Maternal*					
Age (years)	Mean (95%CI)	30.6 (29.9 to 31.2)	30.5 (30.4 to 30.8)	30.4 (29.6 to 31.3)	30.6 (30.4 to 30.8)
Highest level of education	High school or less	20 (10.7)	244 (9.6)	15 (12.2)	240 (9.7)
	Some trade schooling, college or university	23 (12.3)	346 (13.6)	16 (13.0)	329 (13.3)
	Graduated trade school, college, university	111 (59.4)	1,554 (61.1)	75 (61.0)	1,512 (61.1)
	Some graduate school, completed graduate school	33 (17.7)	399 (15.7)	17 (13.8)	394 (15.9)
Household income	<$40,000	23 (12.8)	182 (7.4)	17 (14.4)	181 (7.5)
	$40,000 to <$60,000	24 (13.3)	201 (8.1)	14 (11.9)	200 (8.3)
	$60,000 to <$80,000	24 (13.3)	312 (12.6)	17 (14.4)	293 (12.2)
	$80,000 to <$100,000	24 (13.3)	448 (18.1)	19 (16.1)	427 (17.7)
	≥$100,000	85 (47.2)	1,330 (53.8)	51 (43.2)	1,308 (54.3)
Pre-pregnancy BMI	Underweight (< 18.5 kg/m^2^)	6 (3.2)	116 (4.6)	6 (4.9)	116 (4.8)
	Normal weight (18.5-<25.0 kg/m^2^)	114 (61.6)	1,574 (62.8)	78 (63.9)	1,518 (62.2)
	Overweight (25.0-<30.0 kg/m^2^)	38 (20.5)	553 (22.1)	23 (18.9)	540 (22.1)
	Obese (≥ 30.0 kg/m^2^)	27 (14.6)	265 (10.6)	15 (12.3)	266 (10.9)
Parity	No	97 (52.7)	1,196 (47.2)	74 (60.2)	1,164 (47.2)
	Yes	87 (47.3)	1,337 (52.8)	49 (39.8)	1,300 (52.8)
*Smoking*					
Smoking during pregnancy	No	139 (84.2)	2,085 (89.3)	88 (83.0)	2,030 (89.3)
	Yes	26 (15.8)	249 (10.7)	18 (17.0)	244 (10.7)
Days per week smoked during pregnancy^b^	< 1 day per week	12 (54.6)	144 (60.0)	9 (56.3)	142 (60.9)
	≥ 1 day per week	10 (45.5)	96 (40.0)	7(43.8)	91 (39.1)
Cigarettes smoked per day (on average) during pregnancy^b^	< 1 cigarettes	14 (63.6)	149 (62.3)	11 (68.8)	147 (63.1)
	1–10 cigarettes	8 (36.4)	81 (33.9)	5 (31.3)	76 (32.6)
	> 11 cigarettes	0 (0.0)	9 (3.8)	0 (0.0)	10 (4.3)
Frequency partner smokes	Not at all	146 (79.4)	2,147 (85.3)	93 (76.9)	2,083 (85.1)
	Occasionally	14 (7.6)	148 (5.9)	13 (10.7)	141 (5.8)
	Daily	24 (13.0)	223 (8.9)	15 (12.4)	223 (9.1)
Smoking currently handled	Non-smoking home	129 (71.7)	1,684 (67.5)	82 (71.3)	1,648 (67.8)
	No smoking inside home or with various restrictions	51 (28.3)	810 (32.5)	33 (28.7)	784 (32.2)
Total smoke exposure	None	118 (69.4)	1,841 (78.5)	70 (64.2)	1,790 (78.4)
	Environmental tobacco smoke and/or active smoke	52 (30.6)	503 (21.5)	39 (35.8)	492 (21.6)
*Stress*					
Perceived Stress Scale total score < 25 weeks gestation	Median (IQR)	14 (11 to 19)	13 (9 to 17)	15 (12 to 20)	13 (9 to 17)
Perceived Stress Scale total score 34–36 weeks gestation		14 (9 to 20)	13 (8 to 17)	14 (10 to 20)	13 (8 to 17)
Spielberger State Anxiety Inventory total score < 25 weeks gestation	Median (IQR)	31 (25.5 to 38)	29 (24 to 35)	32 (25 to 41)	29 (24 to 35)
Spielberger State Anxiety Inventory total score 34–36 weeks gestation		36 (29 to 42)	30 (25 to 37)	33.5 (28 to 45)	30 (25 to 37)

95%CI: 95% confidence interval, IQR: Interquartile range

^a^ % unless otherwise specified; ^b^ of those who reported smoking

### Multivariable analyses (no interactions)

Inconsistent results were observed for PSS and STAI scores. Both PSS and STAI scores, in adjusted models, were modestly associated with an increased odds of preterm birth (aOR = 1.03, 95%CI: 1.01–1.06; aOR = 1.03, 95%CI: 1.02–1.05, respectively). STAI but not PSS scores were associated with an increased odds of low birth weight.

Active maternal smoking was associated with a strongly increased odds of preterm birth, though low birth weight did not indicate evidence of an association. Total smoke exposure (active plus environmental tobacco smoke) was also found to be strongly associated with preterm birth but not low birth weight in models without interactions (Tables 2 and 3).

**Table 2:** Logistic regression analyses examining the association between preterm birth or low birth weight, active maternal smoking, total smoke exposure and Perceived Stress Scale scores (complete case and imputed, without interaction, including interaction)

Characteristics	Preterm Birth				Low Birth Weight		
	Adjusted OR (95%CI)^a^				Adjusted OR (95%CI)^b^		
	Imputed without interaction	Complete case^c^ with interaction	Imputed^d^ with interaction		Imputed without interaction	Complete case^c^ with interaction	Imputed^d^ with interaction
Active maternal smoke exposure	1.63 (1.07–2.48)	0.93 (0.25–3.49)	1.36 (0.76–2.43)		1.21 (0.67–2.18)	0.65 (0.25–1.74)	0.89 (0.40–1.97)
Perceived Stress Scale score (PSS)	1.03 (1.01–1.06)	1.03 (1.001–1.06)	1.03 (1.00-1.06)		1.02 (0.99–1.05)	1.01 (0.97–1.05)	1.01 (0.98–1.05)
Interaction: Active maternal smoke exposure and PSS score		1.03 (0.95–1.11)	1.01 (0.98–1.04)			1.03 (0.99–1.08)	1.02 (0.98–1.06)
Total smoke exposure	1.56 (1.07–2.28)	1.62 (0.63–4.17)	1.55 (0.65–3.69)		1.52 (0.93–2.50)	2.64 (0.94–7.40)	2.13 (0.85–5.33)
Perceived Stress Scale score (PSS)	1.03 (1.01–1.06)	1.04 (1.01–1.07)	1.03 (1.01–1.06)		1.02 (0.99–1.05)	1.03 (0.98–1.07)	1.03 (0.99–1.06)
Interaction: Total smoke exposure and PSS score		0.99 (0.94–1.05)	1.00 (0.95–1.05)			0.96 (0.90–1.03)	0.98 (0.92–1.04)

OR: odds ratio; 95% CI: 95% confidence interval

^a^ Adjusted for maternal age, body mass index, education, income, parity; ^b^ Adjusted for maternal age, body mass index, education, income, parity, preterm birth; ^c^ N = 2,154 ^d^ N = 3,341

**Table 3:** Logistic regression analyses examining the association between preterm birth or low birth weight, active maternal smoking, total smoke exposure and Spielberger State-Trait Anxiety Inventory (complete case and imputed, without interaction, including interaction)

Characteristics	Preterm Birth			Low Birth Weight		
	Adjusted OR (95%CI)^a^			Adjusted OR (95%CI)^b^		
	Imputed without interaction	Complete cases with interaction	Imputed with interaction	Imputed without interaction	Complete case with interaction	Imputed with interaction
Active maternal smoke exposure	1.57 (1.02–2.40)	1.54 (0.30–7.87)	1.37 (0.73–2.57)	1.16 (0.64–2.12)	0.58 (0.23–1.49)	0.70 (0.32–1.52)
Spielberger State Anxiety Inventory score (STAI)	1.03 (1.02–1.05)	1.04 (1.02–1.05)	1.03 (1.02–1.05)	1.03 (1.01–1.05)	1.02 (1.00-1.04)	1.02 (1.00-1.04)
Interaction: Active maternal smoke exposure and STAI score		1.00 (0.96–1.04)	1.00 (0.99–1.02)		1.02 (0.999–1.04)	1.02 (1.00-1.03)^*^
Total smoke exposure	1.52 (1.04–2.23)	3.22 (0.93–11.20)	2.31 (0.78–6.82)	1.51 (0.92–2.48)	1.80 (0.40–8.11)	1.47 (0.42–5.11)
Spielberger State Anxiety Inventory score (STAI)	1.03 (1.02–1.05)	1.04 (1.02–1.06)	1.04 (1.02–1.05)	1.03 (1.01–1.05)	1.03 (1.00-1.05)	1.03 (1.00-1.05)
Interaction: Total smoke exposure and STAI score		0.98 (0.94–1.01)	0.99 (0.96–1.02)		0.99 (0.95–1.03)	1.00 (0.97–1.04)

OR: odds ratio; 95% CI: 95% confidence interval

^a^ Adjusted for maternal age, body mass index, education, income, parity

^b^ Adjusted for maternal age, body mass index, education, income, parity, preterm birth

^*^ p = 0.06

### Multivariable analyses (with interactions)

Interaction terms examining total smoke exposure (active maternal plus environmental tobacco smoke), PSS and STAI scores with preterm birth and low birth weight were nonsignificant (Tables 2 and 3). A nonsignificant interaction was also observed when just active maternal smoking and PSS scores were assessed in relation to preterm birth and low birth weight. Findings from the sensitivity analyses including only women who had previously given birth, adjusting for prior preterm birth, were consistent with these results. A marginal association with the interaction of active maternal smoking and STAI scores was observed with low birth weight, after imputation (aOR = 1.02, 95%CI: 1.00-1.03, p = 0.06).

### Multiple linear regression

Active maternal smoking was associated with both PSS and STAI scores in this study. Active maternal smoking was positively associated with PSS scores (adjusted β: 1.10, 95% CI: 0.43–1.78, p-value: 0.001). Active maternal smoking was also observed to be positively associated with STAI scores (adjusted β: 2.27, 95% CI: 1.23–3.30, p-value: <0.001).

## Discussion

In this study, there was no evidence of interactive effects between total smoke exposure and measures of perinatal stress in relation to preterm birth and low birth weight. With imputation, a marginal association between the interaction of active maternal smoking and STAI scores and low birth weight was observed. A positive association was also found between active maternal smoking and STAI scores. This may indicate that certain measures of perinatal stress are linked to the propensity to smoke during pregnancy.

Work is emerging regarding behavioral-psychosocial interactions such as smoking and stress or social support in pregnant women. Lobel et al. [27] found that pregnancy-related stress predicted cigarette smoking, a finding consistent with those in this study. Findings also showed both a direct and indirect (via smoking) relationship with low birth weight [27]. Eisenbruch et al. also observed that among women who experienced low social support, a greater proportion of women reported smoking compared to those receiving high social support (34% vs. 17%, respectively) [18]. Another study from Germany also observed that smokers with low social support were 3.3 times more likely to have a pregnancy complication compared to smokers with high social support [18]. A recent review also observed evidence from several studies that supported an association between perceived stress or number of identified stressors and smoking during pregnancy [43].

Stress during pregnancy may act as a smoking cessation barrier [28, 29]. Bullock et al. [44] investigated the differences between nonsmoking women, women who were successful in quitting smoking during pregnancy, and those who were unable to quit. Findings indicated that differences were observed between the groups in terms of psychosocial stressors, including financial worries, lack of support, and domestic violence [44]. Women who continued smoking during pregnancy were found to have higher levels of stress, lower levels of social support, and were more likely to experience domestic abuse [44]. Thus, the inability to quit smoking during pregnancy may be related to factors beyond just addiction and knowledge deficits [44].

Another hypothesis could be that smoking is a coping mechanism for pregnant women experiencing pregnancy-related anxiety. However, smoking may actually worsen negative emotional states such as stress [45–48]. The perceived relief experienced after smoking may be partially attributable to the alleviation of nicotine withdrawal symptoms rather than a reduction in stress levels [49, 50]. In a U.S.-based randomized trial evaluating different smoking cessation and postpartum relapse approaches, lower perceived stress levels were associated with smoking cessation in early pregnancy but not late pregnancy, [28] which could also have ramifications for the timing of interventions.

Prior studies have observed the modification of the stress-preterm birth/low birth weight relationship by other exposures. Nkansah-Amankra et al. [19] observed this relationship was modified by neighborhood context, with those living in deprived neighborhoods having increased risks of preterm birth and low birth weight. Social environments may affect the risk of preterm birth and low birth weight through stress-related pathways [51]. Similarly, stress and smoke exposure mechanisms may overlap through shared pathways, affecting the risk of adverse birth outcomes. Maternal stress, nicotine, and carbon monoxide in blood may affect fetal and placental development as well as decrease blood flow between the uterus and placenta [23, 52–54]. Both carbon monoxide and stress may also act as a vasoconstrictor of placental blood vessels [23, 52, 53]. Smoke and stress exposure may also affect immunologic functioning and inflammatory responses, leading to adverse birth outcomes [6, 25, 52, 55].

Study findings also suggest that stress, as assessed through Perceived Stress Scale and Spielberger State-Trait Anxiety Inventory scores, are independently associated with an increased odds of preterm birth and/or low birth weight. Both active maternal and total smoke exposure were also observed to be independently associated with preterm birth but not low birth weight in this study. Interactive effects between active maternal smoking, total smoke exposure (active maternal and environmental) and maternal stress were not observed, though active smoking and maternal stress were positively associated.

Our findings were also consistent with other studies that observed an independent positive association between higher maternal stress and preterm birth and low birth weight. Bussières et al. [10] observed in their meta-analysis of prospective studies that prenatal stress was modestly associated with decreased birth weight and shorter gestational age. Very preterm birth and extremely low birth weight exacerbate the risk of neurobehavioral other impairments in children [56, 57].

Data from the 2006 Canadian Maternal Experiences Survey show an estimated prevalence of 10.5% for smoking during pregnancy across Canada, with a reported prevalence of 11.8% in Alberta, [58] which is consistent with the prevalence of smoking in this study (11.6%). Several studies have shown both maternal and environmental tobacco total smoke exposure to be a risk factor for adverse birth outcomes [12–17, 59]. In this study, we found a significant association between both active maternal smoking, total smoke exposure and preterm birth but not low birth weight. In utero smoke exposure (active maternal, environmental tobacco smoke) has also been linked to an increased risk of early adult-onset diabetes and childhood overweight and obesity [60–62].

### Limitations

There are several limitations of the study that must be kept in mind when interpreting these findings. One limitation was the lack of a more refined measure of smoking, which is a recommendation for future work in this area. Information about smoking was also only collected at baseline (< 25 weeks gestation) so smoking habits or exposures may have changed by the time of the second wave of data collection (34–36 weeks gestation). However, prior studies have shown that those who report smoking at the beginning of their pregnancy are likely to continue throughout, though fluctuations in smoking intensity were reported as occurring [63, 64]. Further, the cohort is predominantly white/Caucasian women, which may limit the generalization of these findings to other ethnic groups. Another limitation is the possibility of residual confounding. Sample size and missing data were also other limitations of this study. However, we used a robust method of imputation (with sensitivity analyses) to address this issue. A stress questionnaire specific to pregnant women was not used, though both the PSS and STAI have been used and validated in several other studies [65–70]. As the mechanisms by which stress and smoke exposure may lead to adverse birth outcomes is unknown, the examination of small-for-gestational age is also a potentially important outcome to assess [71, 72].

## Conclusion

Statistically significant interactions between smoke exposure, stress, and anxiety were not observed in this study with preterm birth. A significant interaction between active maternal smoking and Spielberger State-Trait Anxiety in terms of low birth weight was observed only in imputed analyses. Nonetheless, the etiology of adverse birth outcomes points to the role of several factors [6, 7]. The combination of social and behavioral stressors may produce effects beyond either exposure in isolation [18, 19]. Explorations of interactions between environmental, behavioral, and psychosocial exposures can provide important insights into the complex etiology of preterm birth and low birth weight. Critically, in identifying women who experience multiple risk factors, we may also be able to more effectively design and target interventions.

## Data Availability

Study data are available from All Our Families, but restrictions apply to the availability of these data, which were used with permission for the current study and so are not publicly available. Data may be available upon request and with permission from the All Our Families research team (sheila.mcdonald@albertahealthservices.ca).
